# Maternal knowledge and views regarding early hearing detection and intervention in children aged 0–5 years at a semi-urban primary care clinic in South Africa

**DOI:** 10.4102/sajcd.v67i1.681

**Published:** 2020-07-21

**Authors:** Katerina Ehlert, Celeste Coetzer

**Affiliations:** 1 Department of Speech-Language Pathology and Audiology, Faculty of Healthcare Sciences, Sefako Makgatho Health Sciences University, Pretoria, South Africa; 2 Archer & Mann Audiologists Inc., Pretoria, South Africa

**Keywords:** early hearing detection and intervention, maternal knowledge, maternal views, primary care clinic, hearing loss, developing country

## Abstract

**Background:**

South Africans have an increasing burden of hearing loss, especially in low-income rural areas. Limited information is available regarding caregivers’ knowledge and views regarding infant hearing loss, which is essential for the successful implementation of early hearing detection and intervention (EHDI) programmes.

**Objectives:**

The main aim of the study was to describe the knowledge and views of mothers with children aged between 0 and 5 years old regarding hearing screening, risk factors, symptoms of hearing loss, and intervention options for hearing loss.

**Method:**

A survey was employed at a primary care clinic to gain insight into the maternal views on hearing loss and early intervention services for children aged 0–5 years old.

**Results:**

The majority (83.2%) of the mothers believed that hearing can be tested at birth, 90.7% believed in the biomedical model of intervention which is based on cure, prevention, and pathology as opposed to traditional or alternative medicinal beliefs, and 95.3% indicated that they would seek medical attention if they noticed symptoms of hearing loss. Consequences of hearing loss, such as academic, communication and social problems, were indicated by 65.4% of mothers.

**Conclusion:**

The findings demonstrated that although cultural beliefs regarding superstitious causes of hearing loss and use of traditional medicine exist, satisfactory maternal knowledge regarding detection and intervention for hearing loss is present. Maternal views are favourable and a general willingness to participate in EHDI programmes was present. This study advocates for the implementation of EHDI programmes at all primary healthcare clinics across South Africa.

## Introduction

Roughly 141 million live births occurred in the world in 2012, approximately 127 million of which occurred in developing countries (Olusanya, 2012). The estimated prevalence of permanent congenital or early onset hearing impairment in developing countries in 2012 was six cases per 1000 live births. This prevalence is three times higher than in developed countries (Olusanya, 2012). The national prevalence of hearing impairment in South Africa is estimated to be four to six in every 1000 live births in the public health care sector (Maluleke, Khoza-Shangase, & Kanji, 2019).

Primary prevention of hearing impairment, especially in developing countries, is crucial. Secondary and tertiary prevention via early detection and intervention of hearing impairment, especially in infants and young children, are still compulsory and should be actively applied (Olusanya, Neumann & Saunders, 2014). Early hearing detection and intervention (EHDI) services are the foundation for achieving optimal outcomes in infants with hearing loss.

Generally, these services are not widely available or accessible in South Africa. The main goal of EHDI programmes is to promote the early identification and intervention of infants with hearing loss and to identify late onset or progressive hearing loss in children with risk factors who have passed infant hearing screening (Pynnonen et al., 2016). The aim is to ensure optimal and cost-effective solutions that enable effective communication (Health Professions Council of South Africa, 2018). Guidelines for EHDI programmes have been developed in South Africa to ensure optimal outcomes for infants with hearing loss, their families, and society at large (Health Professions Council of South Africa, 2018). The ideal age of identification is 3 months, however in South Africa, the estimated age of identification is between 23 and 31 months (Swanepoel & Almec, 2008; Van der Spuy & Pottas, 2008). The South African public healthcare sector, which serves 85% of the population, only offers EHDI programmes in 7.5% of the hospitals. These statistics differ slightly for the private healthcare sector, with 14% of the hospitals offering EHDI programmes (Casoojee, 2012), and one private hospital group now offering newborn hearing screening as part of their birthing care package (Netcare Limited, 2019). Based on these statistics, less than 10% of South African infants are likely to undergo infant hearing screening.

Infant hearing loss is regarded as one of the most prevalent congenital sensory disabilities affecting double the number of neonates when compared to other screenable disorders impacting the linguistic, educational, socio-emotional and vocational domain (Swanepoel & Almec, 2008). The long-term effects of childhood hearing loss include delayed speech and language development, delayed development of cognitive skills resulting in slow learning and poor academic performance (Peer, 2015), as well as long-term societal costs (Swanepoel & Almec, 2008; Swanepoel, Scheepers, & Le Roux, 2014). Later in life, those with disabling hearing loss experience problems with employment and societal integration (Peer, 2015). The minorities that have jobs are likely to work in lower grades of employment (World Health Organization, 2015). This has a substantial financial implication for South Africa, as the disability grants and unemployment rates increase as a result of late identification and intervention of hearing loss (Mayosi & Benatar, 2014).

Attitudes toward hearing loss are critical to the audiologist’s work; the attitudes of the client, their family, and their community toward the causes, effects and intervention of the hearing loss can be crucial in the rehabilitative process. In many healthcare settings, especially urban locations, the audiologist’s service population is multicultural (Bebout & Arthur, 1992). Attitudes toward disorders are likely to be culture-bound (Bebout & Arthur, 1992). Parental beliefs and perceptions related to disability may influence their knowledge on causes of infant hearing loss and their decisions regarding healthcare and rehabilitation. Parents who believe in the biomedical model (a model of illness that includes biological factors to understand medical illness whilst excluding psychological and social factors) are more likely to consult medical practitioners; and those who believe in traditional/spiritual causes are more likely to seek the services of traditional practitioners (Danseco, 1997).

The biomedical model is based on three concepts, namely, cure, prevention and pathology, therefore health education is based on providing information about diseases, as well as how to cure and prevent them (Carvalho, Dantas & Rauma, 2007). The main idea is to get informed about diseases based primarily on medical research and to persuade individuals to avoid or change unhealthy habits in order to prevent such diseases. In the 20th century, the notion of health promotion emerged, and proposed a more holistic perspective where a person is seen as a bio-psycho-social unit in permanent interaction within him- or herself and his or her environment, including other human beings. The health promotion model considers the biomedical model as only a small part of a larger whole when it comes to the cause of illnesses (Carvalho et al., 2007). The biomedical model views illnesses as a medical entity, whilst the health promotion model views illnesses as an interaction between the individual’s social, psychological and biological environment. Both these models disregard spirituality as a possible cause and/or cure for illnesses.

On the other hand, traditional medicine is defined as diverse health practices, approaches, knowledge and beliefs (Peltzer, 2009). It incorporates plant-, animal- and/or mineral-based medicines, spiritual therapies, manual techniques and exercises. These can be applied singularly or in combination to maintain well-being, as well as to treat, diagnose or prevent illnesses. A study by Astin (1998) revealed that in the United States the majority of alternative medicine users state that they opt for alternative measures because they find these healthcare alternatives to be more congruent with their own values, beliefs and philosophical orientations toward health and life.

In developing countries, public awareness and attitudes towards childhood disabilities such as hearing loss, have been influenced by often irrational customs and beliefs (De Andrade & Ross, 2005; Olusanya, 2000; Stephens, Stephens & Eisenhart-Rothe, 2000). The decisions made by caregivers for infants with hearing loss in relation to EHDI influence the outcomes (Swanepoel, Storbeck & Friedland, 2009). Limited studies regarding mothers’ knowledge and attitudes regarding infant hearing loss in South Africa exist, or they are outdated and specific to a context. More such studies in rural and semi-urban areas, and in a variety of communities, are necessary to gain a nationally representative point of view (Swanepoel & Almec, 2008). Inadequate maternal knowledge of hearing loss is one of the contributing factors to the high number of undetected hearing loss and late implementation of intervention in infants and young children (Swanepoel, Ebrahim, Joseph & Friedland, 2007).

For EHDI to succeed, it is essential to obtain the views of parents regarding infant hearing loss in a variety of contexts in order to identify what practical steps would be required to facilitate its acceptance. This study therefore sought to investigate the maternal views regarding hearing loss in children aged 0–5 years at a primary care clinic in South Africa to gain insights into the prevailing knowledge and attitudes towards EHDI.

## Research methods and design

### Aims

The main aim of the study was to describe the knowledge and views of mothers with children aged between 0 and 5 years old regarding hearing screening, risk factors, symptoms of hearing loss, and intervention options for hearing loss. Secondary objectives were to determine: (1) if the mothers are aware that hearing could be tested and/or screened at birth, (2) what mothers do when they first suspect that their children have a hearing loss, (3) maternal knowledge regarding the consequences and recognition of unidentified and untreated hearing loss, and (4) whether mothers believe in the biomedical model or traditional medicine regarding intervention for hearing loss.

### Participants

#### Inclusion criteria

Inclusion criteria were mothers with children aged 0–5 years, who who were either English, Setswana, Isizulu and Xitsonga proficient, and who were 18 years and older. Exclusion criteria were mothers who had children older than 5 years, who were not literate (unable to read or write), who did not understand English, Setswana, IsiZulu and Xitsonga, and who were younger than 18 years of age.

#### Sample

Purposive sampling was used because this method is based on the judgement of researchers regarding the characteristics of a representative sample (Leedy & Ormrod, 2015). All mothers who met the inclusion criteria and attended the clinic for antenatal care and immunisations for their children were given an equal opportunity to participate.

### Research method and design

A quantitative research approach was employed for the purpose of this study, and a survey (structured questionnaire) was used to conduct the descriptive study.

#### Research site

**FIGURE 1:**
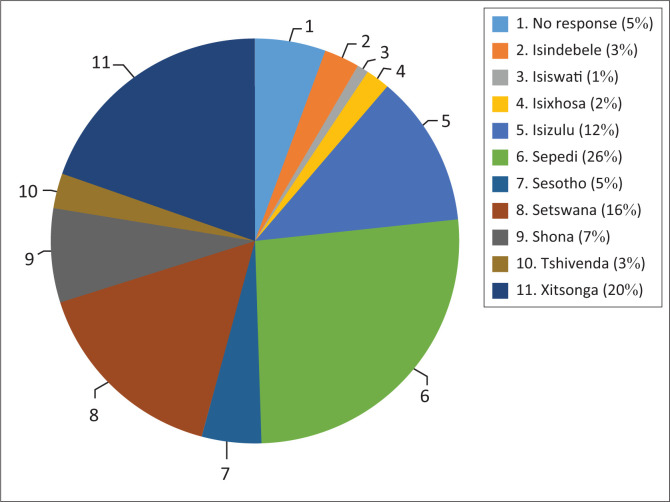
Languages or cultures of participants.

The study was conducted at a primary healthcare clinic in the north of Pretoria, South Africa, that provides EHDI services. The clinic services a large population in the north of Pretoria and provides all primary healthcare services. Although selection of a single clinic may influence the generalisation of results, this clinic services populations from various African backgrounds, as described in Figure 1, which is expected to be similar at other primary care clinics in South Africa. Selection of this site was based on convenience and proximity to researchers.

Speech-Language Pathology and Audiology students have community outreach programmes at the clinic where they educate mothers about the cause, prevention and treatment of hearing loss. There are also audiology services provided at the clinic and the audiologist does biweekly home visits in the community.

#### Questionnaire

A structured questionnaire with open- and closed-ended questions was used. The questionnaire was adapted from focus group questions used in a study in India (Narayansamy, Ramkumar & Nagarajan, 2014). The questionnaire was adapted to ensure that it was contextualised for the South African population, by adding questions appropriate to the South African context. Additionally, options were added to choose from, resulting in more closed-ended questions with an option of providing additional information. The questionnaire consisted of the following sections: demographic data, ear care, causes and prevention of hearing loss in children, hearing assessment, effects and/or signs of hearing loss, and hearing rehabilitation. A pilot study improved the reliability and validity of the questionnaire.

### Data collection

Researchers approached potential participants at the clinic when they attend the clinic for immunisations or antenatal care. Questionnaires were provided to those who met the required inclusion criteria and provided consent. The questionnaires were collected directly after completion. For participants who were unable to read and/or write in English, assistance was provided by the researchers, and verbal translation was provided in their home language.

### Data analysis

Descriptive statistics formed the basis of the data analysis procedure. Answers to the closed-ended questions were easily comparable, coded and analysed. The data collected from the questionnaires were encoded and then tabulated and analysed using Excel for Windows version 10. Averages and means were used to analyse the data. Thematic analysis was used for open-ended questions. Thematic analysis is an innovative research method that combines both a qualitative and quantitative dimension in the study (Meier, Boivin & Meier, 2008). The researchers closely examined the data to identify common themes – topics, ideas and patterns of meaning that came up repeatedly.

### Ethical consideration

Approval was obtained from the Sefako Makgatho Health Sciences Research and Ethics Committee (SMUREC: SMUREC/H/96/2017/UG). Consent was obtained from the Gauteng Department of Health, as well as from the clinic manager before commencement of data collection. For the purpose of the current study, confidentiality and anonymity of respondents were maintained by ensuring that no names appeared on the completed questionnaires.

## Results

The self-administered survey was completed by 107 mothers attending a semi-urban primary healthcare clinic in Tshwane. The minimum age of the mothers in years was 18, and the maximum age was 47. Most mothers (*n* = 102, 95.3%) lived in Soshanguve, 1 (0.9%) came from Hammanskraal and 3.7% (*n* = 4) did not provide a home address.

Figure 1 describes the demographic information for the participants which indicates a multicultural sample including 10 of the 11 languages/cultures represented in South Africa. Sepedi (26%), Xitsonga (20%) and Setswana (16%) were the most prevalent languages and cultures of the participants.

**FIGURE 2:**
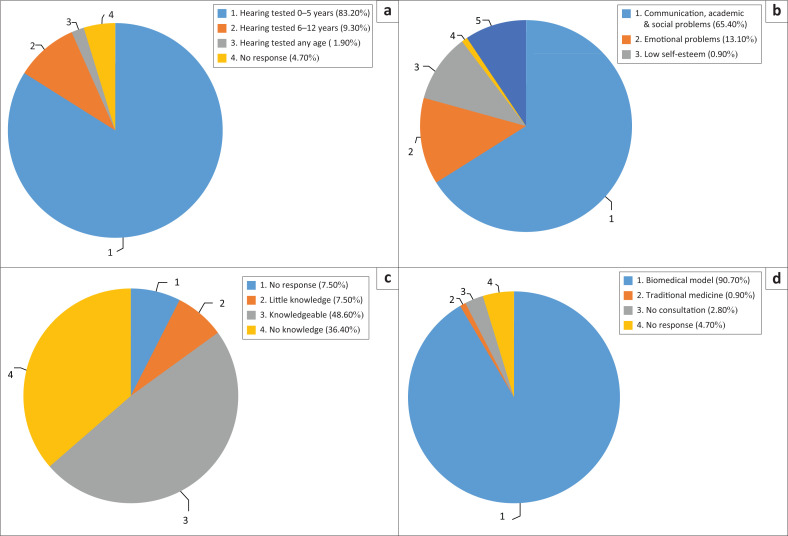
Results according to the objectives of the study: (a) Participants’ beliefs regarding at what age hearing can be tested; (b) participants’ opinions regarding consequences of hearing loss; (c) participants’ knowledge of specialists or professionals involved in ears and hearing; (d) participants’ health beliefs model.

Figure 2 illustrates the main results from the study.

The majority (83.2%) of the mothers reported that hearing can be tested between the ages of 0 and 5 years, whilst 9.3% reported that hearing can be tested at any age.

When mothers were asked what they would do if they suspected that their child has a hearing loss, the overwhelming majority of the participants (95.3%, *n* = 102) would take their child to the clinic, doctor or audiologist.

A limited number of participants (1.9%, *n* = 2) indicated that their child should be taken to the traditional healer and 2.8% (*n* = 3) did not give a response, which could indicate a lack of knowledge or uncertainty in understanding the question even though literacy was an inclusion criterion for this study.

The knowledge of mothers regarding the consequences and recognition of unidentified and untreated hearing loss revealed that the majority of the mothers (62.6%, *n* = 67) indicated that, should a child have normal hearing, the child will look at you when spoken to and 23.4% (*n* = 25) mentioned that the child will startle when hearing a loud sound. Results revealed that most (69.2%, *n* = 74) mothers believed that children will not respond to speech if they have a sign of hearing loss. A few reported that blood or discharge coming out of the ear could indicate hearing loss. A large number of participants (65.4%, *n* = 70) reported that hearing loss can cause communication, academic and social problems and 13.1% (*n* = 14) stated that hearing loss could lead to emotional problems. Only 2.8% (*n* = 3) of the participants indicated that hearing loss can be identified by doing a hearing test.

The reported pre- and post-natal risk factors were prematurity (*n* = 2, 1.9%), infant jaundice and oxygen after birth [*n* = 2, 1.9%] and maternal high blood pressure (*n* = 3, 2.8%). Only five participants (*n* = 5, 4.7%) indicated complications during pregnancy such as bleeding, anaemia, fibroids, placenta praevia and thrombocytopoenia. The remainder of the participants (*n* = 95, 89%) indicated no pre-, peri- or post-natal risk factors or problems.

More than half of the participants (65.4%, *n* = 70) indicated that hearing loss can result from ear malformations or infections, 15.9% (*n* = 17) indicated that it could result from loud noise. Eight (7.5%) of the participants indicated that hearing loss may be a result of punishment from gods or a curse (22.4%, *n* = 24). Furthermore, 2.8% (*n* = 3) indicated both ear malformations or infections and noise as a possible cause of hearing loss. A few (0.9%, *n* = 1) indicated a blocked ear, 0.9% (*n* = 1) indicated putting objects in the ear, and 6.5% (*n* = 7) didn’t respond, which could indicate a lack of knowledge or understanding of the question. When asked if they believe that hearing loss can be a curse, most (64.5%, *n* = 69) indicated no, 22.4% (*n* = 24) indicated yes and 8.4% (*n* = 9) were unsure and indicated maybe; 4.7% (*n* = 5) indicated that they did not know. Almost all participants (90.7%, *n* = 97) indicated that children can be taken to the doctor if they have hearing loss. This supports the notion that most mothers believe in the biomedical model of health as opposed to traditional medicine.

With regard to treatment of hearing loss, most of the participants (72%, *n* = 77) were of the opinion that hearing aids would be an effective treatment option. The second most common treatment option (9.3% (*n* = 10) was aural rehabilitation. The least common treatment option was the use of herbs from a traditional healer (0.9%, *n* = 1). A single participant (0.9%) indicated that all three treatment options could be consulted. Thirteen of the participants (12.1%) indicated that they didn’t know of any treatment options. Most parents (67.3%, *n* = 72) indicated that special schools would be the educational option for a hearing-impaired child. Eighteen participants (16.8%) indicated inclusive schools and 11.2% (*n* = 12) indicated mainstream schools.

In terms of the prevention of hearing loss, the results obtained indicated that the majority (74.8%, *n* = 80) of the mothers indicated that they would regularly visit a doctor or audiologist, followed by avoiding noise exposure (10.3%, *n* = 8), however, it is significant from the results that some (7.5%, *n* = 8) mothers believe that hearing loss can be avoided by praying or giving an offering to the gods and/or ancestors.

## Discussion

The BRICS countries(Brazil, Russia, India, China and South Africa) exhibit substantial and often similar challenges when it comes to the healthcare system and attaining universal health coverage (Marten et al., 2014). This includes, but is not limited to, the inability to provide equal access to the effective healthcare needed, as well as the barriers that exist in accessing healthcare. There is also a shortage and uneven distribution of healthcare workers in these countries (Johl & Pienaar, 2013). The most pressing issues for these five countries when it comes to providing healthcare to its population are: meeting the demands for more human resources; managing changing demographics and disease burdens; and addressing the social determinants of health (Marten et al., 2014). Because of the similarities in the healthcare systems of the BRICS countries, comparisons to the other countries in this group are feasible. Furthermore, the adapted questionnaire was originally used in a study conducted in India, which experiences similar challenges when implementing effective EHDI services.

For developing countries, poor parental compliance as a result of cultural biases to disabilities has been reported (Swanepoel & Almec, 2008). The support and informed choices from parents involved result in a successful EHDI programme. The current study makes an important contribution by revealing parental knowledge and views at a clinic where EHDI services have been provided.

The results of this study indicated that the majority of the parents believed that their children’s hearing can be tested between the ages of 0 and 5 years. Similarly, Swanepoel and Almec (2008) noted that 68% of mothers indicate that hearing loss can be detected soon after birth. The possible explanation for the high percentage in this current study could be that Speech-Language Pathology and Audiology students have community outreach programmes at the clinic, where they educate mothers about the cause, prevention and treatment of hearing loss. There are also audiology services provided at the clinic and the audiologist makes home visits twice a week in the community.

An overwhelming majority of the study participants will take their child to the clinic, doctor or audiologist if they suspect a hearing loss. Govender and Khan (2017) also supported this, but stated that more than 70% of the participants did not know that audiologists are the ones responsible for hearing aid prescription and fitting, as well as aural rehabilitation services. Only 45% of participants were aware that an audiologist is responsible for screening, assessing, diagnosing and managing hearing loss in both children and adults. The audiological services available in this study have promoted the audiology profession, creating awareness amongst the mothers regarding the audiologist’s role.

Regarding the effects of hearing loss on an infant’s development, the results correlate with the study by Narayansamy et al. (2014), where mothers mentioned that the child responds to their name if called when they have normal hearing. However, in their study none of the mothers directly reported the achievement of normal speech milestones as being related to normal hearing, whilst in this current study, mothers mentioned that hearing loss will cause delayed speech and language development. This again could be attributed to the audiological services provided at the clinic and in the community. In this current study, a large number of participants reported that hearing loss can cause communication, academic and social-emotional problems. Similarly, a study in India also reported that most mothers identified lack of learning, difficulty understanding verbal instructions, and inability to lead an independent life as the most frequent effects of hearing loss. A few mothers reported low confidence levels and frustration (Narayansamy et al., 2014).

The reported pre- and post-natal risk factors were prematurity, infant jaundice, oxygen after birth, and maternal high blood pressure. Olusanya, Luxon and Wirz (2006) showed similar results, with good maternal knowledge of the risks related to measles, otitis media, birth asphyxia, and jaundice. It is crucial to educate mothers on various risk factors and management of hearing loss so as to reduce its consequences (Dudda et al.,2017). In South Africa, implementing risk-based hearing screening programmes, which involves screening of newborns with known risk factors for hearing loss, is a more reasonable interim approach to follow in order to ensure early identification of children with hearing loss on the various levels of service delivery contexts within the South African healthcare system (Kanji, 2016).

In developing countries, the use of traditional methods as an alternative measure is rapidly increasing, with Africa having up to 80% of the population using traditional methods to prevent, diagnose and treat illnesses (Peltzer, 2009). A similar study in South Africa by De Andrade and Ross (2005) confirms that eight out of 10 black South Africans consult traditional healers. Traditional healers in South Africa have reported to have been consulted for a variety of audiological and otological problems, and the reason for becoming ill was often sought in a supernatural realm. Most (64.5%) of the participants indicated that hearing loss is not a curse, however some did believe that hearing loss *may* be a curse. Almost all participants indicated that children can be taken to the doctor if they have a hearing loss, again emphasising the belief in the biomedical model.

A study by Govender and Khan (2017), performed in urban KwaZulu-Natal, Durban central region, yielded different results with regard to cultural beliefs. Angry ancestors were noted by 60.8% of the participants and curses by 55.9% as being causes of hearing loss in their infants. The discrepancy in the current study may be related to the audiological services provided in the community used in the current study. A study by Swanepoel and Almec (2008), conducted in the city of Tshwane (urban area), Gauteng, indicated that 44% of mothers have at least one cultural belief (blood impurities) regarding a possible cause of hearing loss. Most parents (82.2%) preferred medical treatment options such as hearing aid fitting and aural rehabilitation, as was found in this study. Only one mother indicated that she would seek alternative options, such as herbs from a traditional healer or prayer. The results, however, also indicated that some mothers prefer using both traditional medicine and biomedical options. De Andrade and Ross (2005) mentioned that treatments for hearing problems used by traditional healers in South Africa may not be effective and may in fact be harmful to patients (e.g. using the insides of the millipede, and sewing machine oil).

The majority of mothers believe in the biomedical model of prevention and treatment of hearing loss. This could be because of the outreach programme that is conducted by students, which educates mothers about hearing loss, causes and treatment options that are available, as well as the audiological services provided at the clinic. Avoiding noise exposure was also reported as a prevention method, however it is significant from the results that some mothers believe that hearing loss can be avoided by praying or giving offerings to the gods and/or ancestors. The proposed explanation is that there is better health awareness in the urban and semi-urban cities. Health awareness is essential in health prevention and improvement, since childhood disabilities could be aggravated as a result of poor public awareness (De Andrade & Ross, 2005; Olusanya, 2001; Olusanya et al., 2006; Swanepoel & Almec, 2008).

The impact on and/or influence of a family’s cultural background should also be considered during assessment and intervention service provision. Barriers regarding cultural competence at each stage of service delivery (during diagnoses, amplification discussions, language assessments, and interventions) have been identified (Grandpierre et al., 2019). Potential linguistic and cultural barriers to interacting with families must be identified and a plan developed to address these needs. This helps to ensure effective communication and follow-up. Culturally- and linguistically-appropriate modes of communication, which may include the use of interpreters or translators, should be used during assessment and management (Geron, 2002). Families should have an active involvement in the assessment and intervention process so that they understand the complex nature of audiologic service delivery. The family’s rights (including informed consent and confidentiality issues), reasonable expectations, reasonable needs and preferences are paramount, and must be considered throughout the provision of services. Additionally, EHDI systems must honour the racial, ethnic, cultural, and socioeconomic diversity of families (American Speech-Language-Hearing Association, 2008). Parental decisions regarding EHDI have dire consequences for the hearing-impaired child. These decisions are often based on parental knowledge and views towards available options regarding infant hearing loss, and are often aggravated by unfavourable superstitious beliefs and customs (Gupta, Sharma & Singh, 2010; Olusanya & Newton, 2007). This situation may delay the promotion of EHDI if it is not addressed appropriately.

The favourable views reported for the communities in this study indicate a general readiness amongst primary caregivers for the improvement of current EHDI programmes and show promising support and cooperation from parents in the primary healthcare context.

### Limitations

The time constraints for the research project contribute to the small sample size. The small sample size was also influenced by the availability of mothers who were willing and qualified to participate in the study. Information pertaining to the educational level of the participants was not included. This could have been beneficial as it could possibly explain why some responses were inappropriate or not completed at all. Furthermore, literacy levels of mothers differed as some mothers (approximately 12%) required assistance when completing the questionnaire, even though they reported being literate. Assistance with completion of the questionnaire could introduce social desirability bias (the desire of respondents to present themselves in the best possible light), however, some research has reported no differences between interviewer versus self-completion modes and type of response (Bowling, 2005). The wide age range of the participants could also have affected the participants’ responses, because older mothers may have more experience and young mothers may have better literacy skills than the older participants. This research study was also context specific and cannot be generalised to the wider population in South Africa. However, 10 languages and/or cultures were represented in this sample, making it representative of the multicultural samples to be expected across primary healthcare clinics in South Africa. Furthermore, the different levels of healthcare in South Africa offer similar services at the primary healthcare level, and this study can serve as a pilot study for investigation of the readiness of mothers to receive EHDI services at other primary healthcare clinics across the province and the country.

### Recommendations for further research

This study was representative of a semi-urban population and results from rural and/or urban populations may be different. Further research is required to determine the readiness of mothers to receive EHDI services in urban, semi-urban and rural primary healthcare clinics across South Africa.

## Conclusion

This study was conducted to gain insight into the knowledge and views of mothers regarding hearing loss, as mothers play a very important role in the health of their children. The results indicated that the majority of the mothers believed that hearing can be tested at birth. An overwhelming number of mothers indicated that they would seek medical attention if they noticed symptoms of hearing loss in their children. A large number of mothers indicated academic, communication and social problems as possible consequences of hearing loss. Most mothers believed in the biomedical model. These results indicate that the mothers have appropriate awareness regarding hearing loss and its management. This could be attributed to the active audiological services in the community regarding prevention, diagnosis, and intervention of hearing loss.

In order for the mothers to make correct and appropriate health decisions for their children, they need to have knowledge about health disorders in their childhood years. Hearing loss is one of the childhood disorders that both mothers and caregivers need to be aware of in light of the high prevalence rates. The current study demonstrated that although cultural beliefs regarding superstitious causes of hearing loss and use of traditional medicine exist, satisfactory maternal knowledge regarding detection and intervention for hearing loss is present. Maternal views are favourable and a general willingness to participate in EHDI programmes was present. This study advocates for the implementation of EHDI programmes at all primary healthcare clinics across South Africa.
